# *Plasmodium falciparum* in the southeastern Atlantic forest: a challenge to the bromeliad-malaria paradigm?

**DOI:** 10.1186/s12936-015-0680-9

**Published:** 2015-04-25

**Authors:** Gabriel Zorello Laporta, Marcelo Nascimento Burattini, Debora Levy, Linah Akemi Fukuya, Tatiane Marques Porangaba de Oliveira, Luciana Morganti Ferreira Maselli, Jan Evelyn Conn, Eduardo Massad, Sergio Paulo Bydlowski, Maria Anice Mureb Sallum

**Affiliations:** Departamento de Epidemiologia, Faculdade de Saúde Pública da Universidade de São Paulo, São Paulo, SP 01246-904 Brazil; Laboratório de Informática Médica (LIM 01), Faculdade de Medicina da Universidade de São Paulo, São Paulo, SP 05405-000 Brazil; Divisão de Doenças Infecciosas, Hospital São Paulo, Escola Paulista de Medicina, Universidade Federal de São Paulo, São Paulo, SP 04024-002 Brazil; Laboratório de Genética e Hematologia Molecular (LIM 31), Faculdade de Medicina da Universidade de São Paulo, São Paulo, SP 05403-000 Brazil; Divisão de Pesquisa, Fundação Pró-Sangue Hemocentro de São Paulo, Faculdade de Medicina da Universidade de São Paulo, São Paulo, SP 05403-000 Brazil; Department of Health, Wadsworth Center, Slingerlands, NY 12159 USA; Department of Biomedical Sciences, School of Public Health, State University of New York-Albany, Albany, NY 12222 USA

**Keywords:** Atlantic forest, *Anopheles*, Forest fragment, Landscape, Malaria, *Plasmodium falciparum*, Transmission dynamics

## Abstract

**Background:**

Recently an unexpectedly high prevalence of *Plasmodium falciparum* was found in asymptomatic blood donors living in the southeastern Brazilian Atlantic forest. The bromeliad-malaria paradigm assumes that transmission of *Plasmodium vivax* and *Plasmodium malariae* involves species of the subgenus *Kerteszia* of *Anopheles* and only a few cases of *P. vivax* malaria are reported annually in this region. The expectations of this paradigm are a low prevalence of *P. vivax* and a null prevalence of *P. falciparum*. Therefore, the aim of this study was to verify if *P. falciparum* is actively circulating in the southeastern Brazilian Atlantic forest remains.

**Methods:**

In this study, anophelines were collected with Shannon and CDC-light traps in seven distinct Atlantic forest landscapes over a 4-month period. Field-collected *Anopheles* mosquitoes were tested by real-time PCR assay in pools of ten, and then each mosquito from every positive pool, separately for *P. falciparum* and *P. vivax*. Genomic DNA of *P. falciparum* or *P. vivax* from positive anophelines was then amplified by traditional PCR for sequencing of the 18S ribosomal DNA to confirm *Plasmodium* species. Binomial probabilities were calculated to identify non-random results of the *P. falciparum*-infected anopheline findings.

**Results:**

The overall proportion of anophelines naturally infected with *P. falciparum* was 4.4% (21/480) and only 0.8% (4/480) with *P. vivax*. All of the infected mosquitoes were found in intermixed natural and human-modified environments and most were *Anopheles cruzii* (22/25 = 88%, 18 *P. falciparum* plus 4 *P. vivax*). *Plasmodium falciparum* was confirmed by sequencing in 76% (16/21) of positive mosquitoes, whereas *P. vivax* was confirmed in only 25% (1/4). Binomial probabilities suggest that *P. falciparum* actively circulates throughout the region and that there may be a threshold of the forested over human-modified environment ratio upon which the proportion of *P. falciparum*-infected anophelines increases significantly.

**Conclusions:**

These results show that *P. falciparum* actively circulates, in higher proportion than *P. vivax*, among *Anopheles* mosquitoes of fragments of the southeastern Brazilian Atlantic forest. This finding challenges the classical bromeliad-malaria paradigm, which considers *P. vivax* circulation as the driver for the dynamics of residual malaria transmission in this region.

**Electronic supplementary material:**

The online version of this article (doi:10.1186/s12936-015-0680-9) contains supplementary material, which is available to authorized users.

## Background

The current paradigm of malaria epidemiology in the Brazilian Atlantic forest is based on the bromeliad-malaria model proposed by Lutz [1], Deane [2] and Gadelha [3], among others (e.g., [4]). The bromeliad-malaria hypothesis proposes that malaria in humans is caused by interactions between mosquito vectors of the subgenus *Kerteszia,* genus *Anopheles* (*Anopheles cruzii* as a primary vector), and *Plasmodium vivax* and *Plasmodium malariae* pathogens [4-6]. Bromeliad-malaria also includes simian malaria, a related cycle in which *An. cruzii* can be infected by and transmit *Plasmodium simium* to howler monkeys (*Aloutta spp*.) [2,7]. Mosquito species included in the subgenus *Kerteszia* are adapted to bromeliad phytotelmata as habitats for their immature stages [8]. Consequently, humans in close contact with tropical rain forests with abundant bromeliad vegetation can be exposed to infective bites from females of those mosquitoes [9,10].

Epidemics of malaria during the 1940s and 1950s in the southeastern Atlantic forest of Brazil have been primarily associated with the dynamics of bromeliad-malaria. Transmission was successfully controlled and malaria incidence decreased to a hypo-endemic level by an aggressive vector control program that included complete deforestation of areas where the incidence of the disease was high and *Kerteszia* species were the primary vectors. This massive effort to destroy bromeliads diminished the abundance of *Kerteszia* mosquitoes and eliminated the burden of malaria on humans [11]. Since that period, malaria has become residual with a very low level of transmission (Annual Parasite Index [API] <0.1), and few autochthonous annual cases reported [12]. This situation led the Brazilian Ministry of Health to declare malaria non-endemic status for areas within the Atlantic forest after the 1970s [13,14]. However, residual malaria outbreaks in several localities within this biome [12,15] motivated investigations focused on the mosquito and parasite species associated with malaria transmission [10,16-20]. These research groups detected the involvement of species of the *Kerteszia*, *Nyssorhynchus* and *Anopheles* subgenera of genus *Anopheles* as vectors [10,20], and *Alouatta* and *Cebus* monkeys as potential reservoirs [16-19]. The high frequency of reactions against the repetitive epitopes of the circumsporozoite protein (CSP) of *Plasmodium falciparum* and *P. vivax* suggests that the infection of non-human primates [16,17] by these *Plasmodium* species has been neglected. Taken together, these results indicate that the bromeliad-malaria hypothesis, which does not encompass the potential circulation of *P. falciparum* in areas of Atlantic forest, needs to be re-evaluated.

Recently, a cross-sectional study revealed a surprisingly high frequency (5.14%, 57/1,108) of *P. falciparum* real-time PCR positivity in asymptomatic blood donors living or in close contact with forested regions of the southeastern Brazilian Atlantic forest biome [21]. The bromeliad-malaria paradigm would predict no *P. falciparum* infection and very low frequency of *P. vivax* infection in asymptomatic blood donors inhabiting forested areas of São Paulo state [22]. In fact, Mendrone *et al*. [22] suggested that the *P. falciparum* real-time PCR positivity found in asymptomatic blood donors [21] could be an artifact. However, another study proposed that alternative dynamics of *Plasmodium* transmission may have evolved and caused the unexpected high frequencies of *P. falciparum* DNA in humans, the high level of antibodies against CSP in monkeys and *Plasmodium*-infected mosquito species other than *Kerteszia* [23]. The present study searched for *P. falciparum* circulating in mosquitoes captured in forest fragment areas within the Atlantic forest domain in southeastern São Paulo state. The major objectives of this study were to: 1) address the occurrence of *Anopheles* infection by *P. falciparum* and/or *P. vivax*; and, 2) verify the range of *P. falciparum* circulation in the biome, scrutinizing possible relationships between frequency of *P. falciparum* infection in anophelines and different grades of intermixed natural and human-modified environments.

## Methods

### *Anopheles* mosquito collection

**Figure 1:**
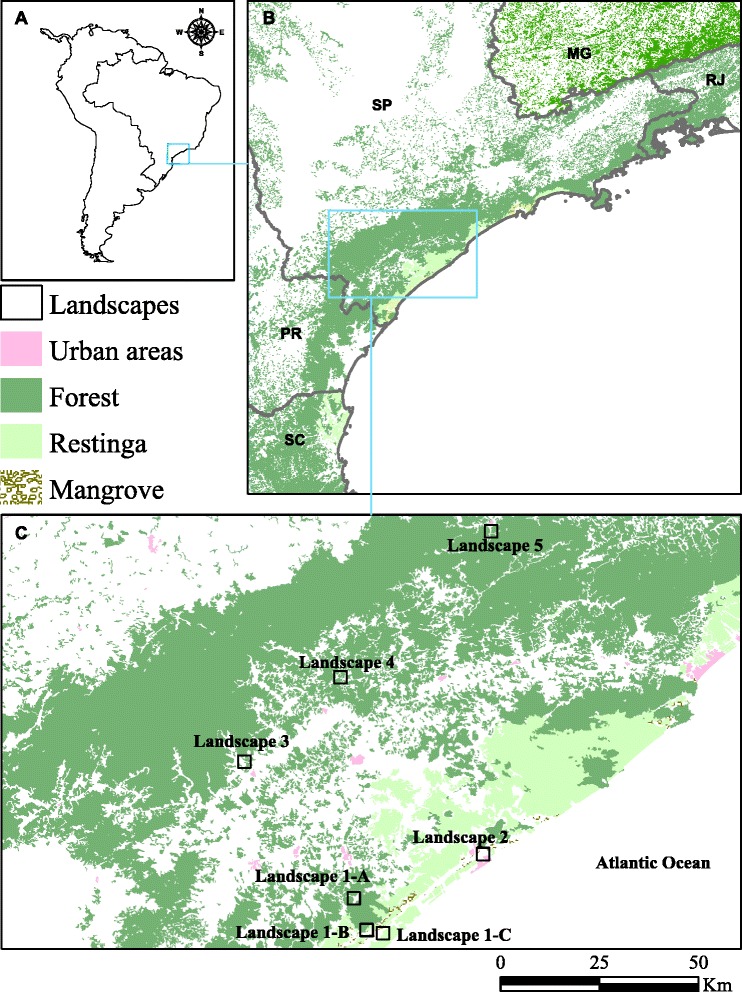
Study region and design. **A)** South America. **B)** Southeastern Brazilian Atlantic forest. SP: São Paulo state, MG: Minas Gerais state, RJ: Rio de Janeiro state, PR: Paraná state, SC: Santa Catarina state. **C)** Landscapes 1–5 (10 sq km) representing spatial scale in which dynamics of malaria transmission can occur, given the estimated home ranges of vectors and parasites. Source: SOS Mata Atlântica, Instituto Nacional de Pesquisas Espaciais (INPE), 2008.

Field collections were conducted in seven localities situated within an area of 11,000 sq km in the fragmented remains of the Atlantic forest in southeastern São Paulo state. To sample mosquitoes, a landscape-based, cross-sectional design was adopted as follows: landscapes 1-B and 1-C – predominantly natural vegetation; landscapes 1-A, 3, 4, and 5 – natural vegetation intermixed with rural and urban areas; landscape 2 – predominantly human-modified environment. The characterization of features (e.g., natural vegetation, open areas) in each landscape was performed by supervised image classification technique in ArcGIS 10 Spatial Analyst™ Image Classification tool. Accordingly, a known setting of trained pixels was applied in order to convert multiband Landsat 5 TM into a single band image with different categories of landscape features. The conversion was based on differential spectral sunlight responses to distinct landscapes. Forest fragments in multiband Landsat 5 TM images showed low pixel values in blue and red bands, because of the high absorption in these wavelengths for photosynthesis. On the contrary, forest reflectance was high for green and near infrared bands. Exposed soil in rural areas had low pixel values for blue, green and red bands, whereas water had high pixel values for the blue band only. Urban areas had a mixture of reflectance and combinations of spectrally distinct land cover categories. The proportion of each land cover type (i.e., natural vegetation, rural and urban areas) within each landscape was estimated with the aid of FRAGSTATS version 4 [24]. In Figure 1, dark green areas correspond to forested areas; light green areas correspond to Atlantic coast restinga; and tiny brownish areas correspond to mangrove. White areas correspond to human modified environments and pink areas represent urban areas. See Figure 1 for details.

Mosquitoes were captured from August to November 2012, using CDC-LT (Centers for Disease Control light traps), with and without Octenol plus CO_2_ as attractants, and Shannon traps. The location of traps in the field was planned to capture mosquitoes in transition zones (forest fringes and forest edges), inside forest fragments (i.e., patches of conspicuous natural vegetation) and in anthropogenic areas (i.e., either rural or urban), in each landscape.

**Figure 2:**
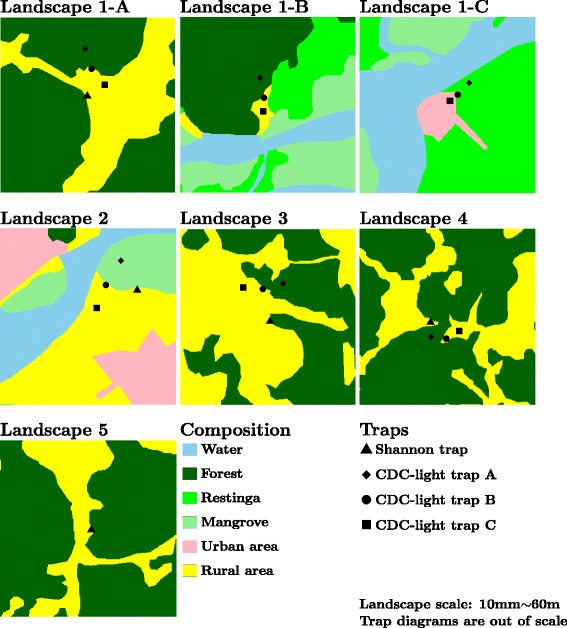
Landscape features and trap locations. Landscape 1-A – Esteiro do Morro, natural vegetation (65.37%) and rural (34.63%). Landscape 1-B – Taki, natural vegetation plus water (97.49%) and rural (2.51%). Landscape 1-C – Pedrinhas, natural vegetation plus water (94.48%) and urban (5.52%). Landscape 2 – Boqueirão Norte, natural vegetation plus water (38.94%), rural (38.90%) and urban (22.16%). Landscape 3 – Eldorado, natural vegetation (44.66%) and rural (55.34%). Landscape 4 – Sete Barras, natural vegetation (65.37%) and rural (34.63%). Landscape 5 – Tapiraí, natural vegetation (74.99%) and rural (25.01%). CDC-light trap A: one CDC-LT with attractants and one CDC-LT 1.5-m above the ground and one CDC-LT in the tree canopy; CDC-light trap B: one at the level of the tree canopy and one 1.5-m above the ground; CDC-light trap C: one CDC-LT 1.5-m above the ground.

Six CDC traps were distributed in three localities in each landscape: a) approximately 50 meters inside the forest; b) at the forest edge; and c) in the open area approximately 50 meters from the forest edge. See Figure 2 for details of the trap locations in each landscape.

Specifically, three CDC-LT were placed inside the forest: one CDC-LT with Octenol plus CO_2_ and one CDC-LT without attractant were installed 1.5 metres above the ground (~20 metres apart) and one in the tree canopy. Two CDC-LTs without attractant were installed at the forest edge: one at the level of the tree canopy and one 1.5 metres above the ground. Finally, one CDC-LT without attractant was installed in the open area, 1.5 metres above the ground. For all CDC-LT traps the period of sampling extended from 18:00–06:00 h, when they were collected for analysis (see Additional files 1 and 2 for details). This sampling procedure was repeated the following day in each landscape, totaling 12 CDC-LTs and 144-hour-effort. In landscapes 1-B and 1-C only CDC-LT traps were used.

Additionally, collections with Shannon traps were performed from 17:00–05:00 h, totaling 12-hour-effort, in landscapes 1-A, 2, 3 and 4. In landscape 5, Shannon trap was used in a three-hour-effort, from 17:00–20:00 h (see Additional files 1 and 2 for details).

### *Anopheles* identification

Each mosquito was morphologically identified using a standard dichotomous key [25]. A subsample of individuals, from each species collected, was identified by DNA sequencing of the *COI* barcode region of the mitochondrial DNA genome, for morphological identification confirmation, according to Foster *et al*. [26] protocols. Homology between the *COI* mtDNA sequences from *Anopheles* individuals generated in this study and those available in GenBank was assessed using the nucleotide BLAST search algorithm [27]. Template DNA from both non-infected *Anopheles* species and *Anopheles* infected with *Plasmodium* spp. was stored at −70°C in a reference entomological collection, the Coleção Entomológica de Referência, Universidade de São Paulo, Brazil, for future reference.

### Genomic DNA extraction

DNA was extracted from each *Anopheles* specimen individually using a salting-out method [28]. The head and thorax of each *Anopheles* individual was separated from the abdomen using a fine entomological pin as suggested by Foley *et al*. [29]. Each head and thorax was then mixed in 500 μL TEN buffer (2 mM Tris–HCl, pH 8.0, containing 0.5 mM EDTA and 5 mM NaCl) with 5 μL 10% SDS (sodium dodecyl sulphate) and 3-μL proteinase K (20 mg/mL). The mixture was homogenized with a tissue tearor (model 985370–395 Biospec products) until complete insect disruption and incubated for 1 hr at 56°C. Then, 150 μL saturated NaCl (5 M) was added and the mixture was stirred vigorously for 30 sec. After centrifugation at 5,000 rpm for 10 min at room temperature (18-25°C), the supernatant was transferred to a 1.5-ml plastic vial containing 600 μL of cold isopropanol. This tube was inverted several times to aid DNA precipitation, and was kept at -20°C overnight to increase precipitation. The tube containing DNA was spun at 12,000 rpm for 10 min at 4°C. The pellet was then washed four times with 70% ethanol, and centrifuged at 5,000 rpm for 5 min at room temperature to remove any excess salt. DNA was dried for 20 min in a vacuum centrifuge AES1010 Speed Vac (Savant, USA), and resuspended in 20 μL TE (2 mM Tris–HCl, pH 8.0, 0.5 mM EDTA). The total amount of genomic DNA obtained from each individual mosquito head and thorax was quantified using a NanoDrop (ND-1000 UV–vis Spectrophotometer).

### Real-time PCR assay by hydrolysis probe

A standardized real-time PCR protocol adapted from [30] and previously used in our laboratory [21] was employed to detect *P. falciparum* and *P. vivax* DNA in *Anopheles* specimens. The specificity and sensitivity of this protocol was verified utilizing a 10-fold serial dilution of different positive controls: 1) a laboratory-culture of *P. falciparum* to test the specificity of the hydrolysis probe; 2) a blood sample obtained from a patient infected with *P. vivax* to test the specificity of the hydrolysis probe, 3) genomic DNA extracted from one head plus thorax of *Anopheles gambiae* infected with *P. falciparum* to test the sensitivity of the hydrolysis probe and, finally, 4) a mixture of DNA obtained from a patient with *P. vivax* and genomic DNA extracted from the head and thorax of one non-infected *Anopheles* mosquito (adult emerged in laboratory from field collected pupa) to test the sensitivity of the hydrolysis probe. A PCR amplification product was obtained only when specific *Plasmodium* species were present in the reaction; no product was detected in samples without specific parasites, or when *Anopheles stephensi* infected with *Plasmodium berghei* was tested. The lack of cross-amplification between different reactions demonstrated that each was specific for the *Plasmodium* species tested.

The samples were tested in pools of genomic DNA from 10 *Anopheles* individuals. Each pool was made with 100 ng of DNA extracted from each mosquito and the real-time PCR test used 300 ng of the pooled DNA. After this, each mosquito from a positive pool was individually retested using 30 ng of DNA. Samples of laboratory-cultured *P. falciparum* and of human blood samples positive for *P. vivax* were used as positive controls for the real-time PCR reactions. Each sample was tested in duplicate and positive samples were retested in two distinct real-time PCR reactions. Each real-time PCR reaction had a final volume of 12.5 μL with 5 μL from the final ddH_2_O diluted field sample, 6.25 μL of the TaqMan™ Gene Expression Master Mix (Applied Biosystems, Foster City, CA, USA) and 1.25 μL of 300 nM forward primer, 300 nM reverse primer for either *P. vivax* or *P. falciparum* and 200 nM of TaqMan**™** probe. Primers and TaqMan**™** probes are described elsewhere [21,30]. Real-time PCR amplification was achieved using a 7500 FAST real time PCR system (Applied Biosystems, Foster City, CA, USA) with a programme involving two thermal cycler holds. Conditions were 50°C for 2 min and 95°C for 10 min, followed by 50 cycles of amplification (95°C for 15 sec and 60°C for 1 min). Results were analyzed using the 7500 software v.2 0.5 (Applied Biosystems, Foster City, CA, USA).

### *Plasmodium* PCR amplification and DNA sequencing

To confirm the presence of either *P. falciparum* or *P. vivax* DNA in *Anopheles* mosquitoes, the genomic DNA mix (mosquito plus *Plasmodium* DNA) obtained from the head and thorax of each field-collected mosquito positive in the real-time PCR was checked separately, employing a single template PCR amplification strategy. Each DNA mix was tested only for the *Plasmodium* species identified in the real-time PCR. PCR amplification was carried out using the same set of species-specific primers employed for the real-time PCR, without the TaqMan**™** probes. The pairs of primers FAL-F (5′ CTTTTGAGAGTTTTGTTACTTTGAGTAA 3′), FAL-R (5′ TATTCCATGCTGTAGTATTCAAACACAA 3′) were used to amplify a ~96 base pair fragment of the 18S rDNA region of *P. falciparum*, and the pair VIV-F (5′ ACGCTTCTAGCTTAATCCACATAACT 3′), VIV-R (5′ ATTTACTCAAAAGTAACAAGGACTTCCAAGC 3′) amplified a ~140 base pair fragment of *P. vivax*. The PCR was performed in a final volume of 25 μL containing 1 μL DNA extraction solution, 12.5 μL reagent TaqMan**™** Gene Expression Master Mix (Applied Biosystems, Foster City, CA, USA) with 0.3 μM forward primer and 0.3 μM reverse and the remaining volume of ddH_2_O. The PCR thermal conditions were the same as those employed for the real-time PCR. The PCR products were purified by DNA Clean & Concentrator™ kit (Zymo Research), and sequenced in both directions, using Sanger technology [31], with the same set of primers employed for amplification. Sequencing reactions were carried in a total volume of 10 μL containing 20 ng of the purified PCR product, 0.5 μL BigDye™ Terminator Ready Reaction Mix, 2 μL of 1× Sequencing Buffer (Applied Biosystems), 3.6 pmol of reverse or forward primers and the remaining volume of ultrapure H_2_O. Sequencing reactions were purified in Sephadex G50 columns (GE Healthcare), analyzed on an ABI Prism 3130 - Avant Genetic Analyzer (Applied Biosystems, Foster City, CA, USA), and edited using Sequencher™ version 5.1 (Gene Codes Corporation, Ann Arbor, USA).

### Binomial test

In order to detect non-random outcomes of the obtained results per landscape, it was performed a binomial distribution analysis with the anopheline-*P. falciparum* infection data. Considering the overall prevalence of anopheline-*P. falciparum* infection obtained in the present study (see Results) as the *a priori* probability, two probabilities were calculated assuming the binomial distribution. First, it was calculated the probability of having at least one positive anopheline in the sample obtained. From this probability, the statistical power (1 - β) of each sample in detecting *P. falciparum* infection in the corresponding landscape, or species, or trap was estimated. Then, it was calculated the exact probability of finding the number of positive mosquitoes (k) among the total tested (n) per landscape, species, or trap. If this probability was under the adopted level of significance then it was used as evidence of a non-random outcome, i.e., below or above the expected result (i.e., the aforementioned overall prevalence). The significance level of 0.05 and the power of the test of 0.8 were adopted [32].

## Results

A total of 921 *Anopheles* specimens were captured. A subsample of 480 anophelines (over the 921 captured) was used to the real-time PCR testing. The subsample selection strategy was oriented to proven vectors in the region (e.g., *An. cruzii*, *An. bellator* and *An. marajoara*) [33] plus others that were found infected recently (e.g., *An. strodei*, *An. triannulatus*, *An. fluminensis*) [10,20] or other abundantly captured species (e.g., *An. galvaoi*, *An. mediopunctatus*). Table 1 shows the absolute and relative frequencies of *Anopheles* species captured.

**Table 1:** **Absolute (n) and relative frequency (%) of field collected** ***Anopheles*** **specimens per landscape, Atlantic forest, Brazil, August-November 2012**

**Landscape**	**Traps**	**Species**	**Frequency**		**Period of collection**
			**n**	**%**	
1-A	Shannon	*An.* (*Ano.*) *fluminensis* ^a^	4	0.43	Mid October
	CDC-LT	*An.* (*Ano.*) *intermedius*	2	0.22	Late August
	Shannon	*An.* (*Ano.*) *intermedius* ^a^	12	1.30	Mid October
	CDC-LT	*An.* (*Ker.*) *bellator*	1	0.11	Late August
	CDC-LT	*An.* (*Ker.*) *cruzii*	2	0.22	Late August
	Shannon	*An.* (*Ker.*) *cruzii* ^a^	4	0.43	Mid October
	Shannon	*An.* (*Nys.*) *strodei* s.s.^a^	100	10.86	Mid October
	Shannon	*An.* (*Nys.*) *triannulatus* ^a^	21	2.28	Mid October
Subtotal			146	15.85	
1-B	CDC-LT	*An.* (*Ker.*) *bellator*	5	0.54	Mid August
	CDC-LT	*An.* (*Ker.*) *cruzii*	27	2.94	Mid August
Subtotal			32	3.48	
1-C	CDC-LT	*An.* (*Ano.*) *mediopunctatus*	19	2.06	Mid August
	CDC-LT	*An.* (*Ker.*) *cruzii*	1	0.11	Mid August
Subtotal			20	2.17	
2	Shannon	*An.* (*Ano.*) *intermedius* ^a^	1	0.11	Mid September
	CDC-LT	*An.* (*Ano.*) *mediopunctatus*	12	1.30	Late August
	Shannon	*An.* (*Ano.*) *mediopunctatus* ^a^	8	0.87	Mid September
	Shannon	*An.* (*Nys.*) *albitarsis* s.s.^a^	14	1.52	Mid September
Subtotal			35	3.80	
3	Shannon	*An.* (*Ano.*) *intermedius*	14	1.52	Late October
	CDC-LT	*An.* (*Ano.*) *mediopunctatus*	2	0.22	Late August
	Shannon	*An.* (*Ano.*) *mediopunctatus* ^a^	51	5.54	Late October
	CDC-LT	*An.* (*Ker.*) *cruzii*	1	0.11	Late August
	Shannon	*An.* (*Ker.*) *cruzii*	19	2.06	Late October
	Shannon	*An.* (*Nys.*) *albitarsis* s.s.^a^	2	0.22	Late October
	Shannon	*An.* (*Nys.*) *galvaoi* ^a^	147	15.96	Late October
	Shannon	*An.* (*Nys.*) *oswaldoi* s.l.	13	1.41	Late October
	Shannon	*An.* (*Nys.*) *triannulatus*	14	1.52	Late October
Subtotal			263	28.56	
4	Shannon	*An.* (*Ano.*) *intermedius*	2	0.21	Mid September
	CDC-LT	*An.* (*Ano.*) *mediopunctatus*	1	0.11	Late August
	Shannon	*An.* (*Ano.*) *mediopunctatus* ^a^	1	0.11	Mid September
	CDC-LT	*An.* (*Ker.*) *cruzii*	1	0.11	Late August
	Shannon	*An.* (*Ano.*) *mediopunctatus* ^a^	3	0.32	Mid September
	Shannon	*An.* (*Nys.*) *albitarsis* s.s.	14	1.52	Mid September
	Shannon	*An.* (*Nys.*) *galvaoi* ^a^	58	6.30	Mid September
	Shannon	*An.* (*Nys.*) *oswaldoi* s.l.	8	0.87	Mid September
	Shannon	*An.* (*Nys.*) *triannulatus*	39	4.24	Mid September
Subtotal			127	13.79	
5	Shannon	*An.* (*Ker.*) *cruzii* ^a^	251	27.25	Late November
	Shannon	*An.* (*Nys.*) near pristinus	4	0.43	Late November
	Shannon	*An.* (*Nys.*) *strodei* s.s.^a^	40	4.34	Late November
	Shannon	*An.* (*Nys.*) *triannulatus*	3	0.33	Late November
Subtotal			298	32.35	
Total			921	100	

^a^Morphological identification confirmed by DNA *COI* barcode sequences.

Twenty-five specimens were infected with *Plasmodium* parasites, for an overall prevalence of 5.2% (25/480). As related to the malaria parasites, 21 out of 25 (84%) were positive for *P. falciparum*, contrasting with only four out 25 (16%) which were positive for *P. vivax*. None of the mosquito samples was tested for *P. malariae,* in spite of the traditional acknowledgement of this species participation in the bromeliad-malaria paradigm. Nevertheless, between 1980 and 2007, only five cases were attributed to *P. malariae* (less than 1%, 5/821 cases) in São Paulo state [34].

*Anopheles cruzii* was the species with the highest infectivity (22/260, 8.5%), and represented almost the totality of infected anophelines found (22/25, 88%). There was a preponderance of *P. falciparum* infection among them (18/22, 82%, of *P. falciparum* and 4/22, 18%, of *P. vivax*). The remaining three anophelines were infected with *P. falciparum*, being one *An. strodei*, one *An. triannulatus*, and one *An. galvaoi*. Table 2 shows the amount of anophelines tested and positive in each landscape studied.

**Table 2:** ***Anopheles*** **species, number of mosquitoes tested positive for** ***Plasmodium*** **DNA/number of individuals tested for infection (+/n), frequency (%) of infected** ***Anopheles*** **, PCR method (real-time and conventional PCR) and the parasite species per landscape, Atlantic forest, Brazil, August-November 2012**

**Landscape**	**Species** ^**a**^	**Real-time PCR +/n (%)**	**PCR +/n (%)**	**Parasite species** ^**b**^
1-A	*An. cruzii*	0/6 (0.0)	-	-
	*An. fluminensis*	0/3 (0.0)	-	-
	*An. strodei*	1/72 (1.4)	0/1 (0.0)	*P. falciparum*
	*An. triannulatus*	1/12 (8.3)	0/1 (0.0)	*P. falciparum*
	Subtotal	2/93 (2.15)	0/2 (0.0)	-
1-B	*An. cruzii*	0/22 (0.0)	-	-
	*An. bellator*	0/9 (0.0)	-	-
	Subtotal	0/31 (0.0)	-	-
1-C	*An. cruzii*	0/1 (0.0)	-	-
	Subtotal	0/1 (0.0)	-	-
2	*An. albitarsis*	0/14 (0.0)	-	-
	Subtotal	0/14 (0.0)	-	-
3	*An. albitarsis*	0/1 (0.0)	-	-
	*An. cruzii*	0/20 (0.0)	-	-
	*An. galvaoi*	1/48 (2.1)	1/1 (100.0)	*P. falciparum*
	*An. mediopunctatus*	0/42 (0.0)	-	-
	Subtotal	1/111 (0.9)	1/1 (100.0)	-
4	*An. albitarsis*	0/13 (0.0)	-	-
	*An. cruzii*	0/4 (0.0)	-	-
	*An. strodei*	0/1 (0.0)	-	-
	*An. triannulatus*	0/5 (0.0)	-	-
	Subtotal	0/23 (0.0)	-	-
5	*An. cruzii*	4/207 (1.9)	1/4 (25.0)	*P. vivax*
	*An. cruzii*	18/207 (8.7)	15/18 (83.0)	*P. falciparum*
	Subtotal	22/207 (10.6)	16/22 (72.7)	-
	Total *P. vivax*	4/480 (0.8)	1/4 (25.0)	
	Total *P. falciparum*	21/480 (4.4)	16/21 (76.2)	
	Total	25/480 (5.2)	17/25 (68.0)	

^a^Morphological identification of the specimens tested positive for *Plasmodium* was confirmed by DNA *COI* barcode sequences.

^b^ *Plasmodium* species identified in Anopheles mosquitoes by real-time PCR and/or conventional PCR plus DNA sequencing with species-specific primers [21].

Table 2 shows that eight out of the twenty-five anopheline samples that tested positive with real-time PCR for *Plasmodium* did not amplify parasite DNA (3 *P. vivax* and 5 *P. falciparum*) in conventional PCR (Additional file 3). From these 17 samples, 16 (15 of *P. falciparum* and 1 of *P. vivax*) had their fragment of the 18S rDNA gene sequenced and submitted to BLAST analysis. The *P. falciparum* fragments shared 99% similarity with *P. falciparum* sequences previously associated with wild monkeys (*Lagothrix cana cana*, KC906727 and *Alouatta puruensis*, KC906718; [35]) and 98% similarity with *Callicebus brunneus* (KC906722; [35]), from the Amazon region of Brazil. In contrast, the 18S rDNA sequence of *P. vivax* shared 100% similarity with two samples sequenced from human isolates in India (JQ627157 and JQ627156). Morphological identification of the 25 anophelines positive for *Plasmodium* infection was confirmed using DNA sequences of the barcode region of the *COI* mitochondrial gene (GenBank accession numbers in Additional file 4).

The distribution of infected mosquitoes was neither spatially nor taxonomically random. Table 3 shows the results of the specific binomial probabilities of positive findings for landscapes, *Anopheles* species, and traps.

**Table 3:** **Analysis of the binomial probability distribution of anopheline-** ***P*** **.** ***falciparum*** **infection according to landscape or species or traps, Atlantic forest, Brazil, August-November 2012**

**Landscape**	**Positive (k)**	**Tested (n)**	**Probability (P)** ^**a**^	**1 – P (k = 0)**
1-A	2	93	0.138	0.985
1-B	0	31	0.248	0.752^c^
1-C	0	1	0.956	0.044^c^
2	0	14	0.533	0.467^c^
3	1	111	0.035^b^	0.993
4	0	23	0.355	0.645^c^
5	18	207	0.003^b^	0.997
**Species**	**Positive (k)**	**Tested (n)**	**Probability (P)** ^**a**^	**1 – P (k = 0)**
*An. albitarsis*	0	28	0.284	0.716^c^
*An. bellator*	0	9	0.667	0.333^c^
*An. cruzii*	18	260	0.018^b^	0.999
*An. fluminensis*	0	3	0.874	0.126^c^
*An. galvaoi*	1	48	0.255	0.885
*An. mediopunctatus*	0	42	0.151	0.849
*An. strodei*	1	73	0.126	0.963
*An. triannulatus*	1	17	0.364	0.535^c^
**Traps**	**Positive (k)**	**Tested (n)**	**Probability (P)** ^**a**^	**1 – P (k = 0)**
CDC-LT	0	38	0.181	0.819
Shannon	21	442	0.083^b^	1

^a^An *a priori* probability of success (k/n) in each trial equals to 0.044, which was the overall prevalence of anopheline-*P. falciparum* infection obtained herein (21 /480), was assumed.

^b^This result was statistically significant (level of significance < 0.05).

^c^This result shows that the statistical power was low (<0.80).

From Table 3, it can be seen that in 3/7 different landscapes *P. falciparum*-infected anophelines were found, for an overall prevalence of 4.4% (21/480). However, it was not possible to exclude active *P. falciparum* circulation all over the region, as the power of the test was consistently below the threshold of 80% for the negative landscapes. In addition, in landscape 5 (natural vegetation intermixed with rural areas, in a ratio 3:1) the prevalence of infected anophelines found was higher than expected. On the other hand, in landscape 3 (natural vegetation intermixed with rural areas, in a ratio 0.8:1), a more intensively human modified environment, the prevalence of infected anophelines found was lower than expected. Finally, in landscape 1-A (natural vegetation intermixed with rural areas, in a ratio 1.9:1), representing an intermediary forest covered environment comparing to landscapes 5 (more forested) and 3 (less forested), the prevalence of infected anophelines found was not significantly apart from the expected overall prevalence for the region. These findings could suggest that there may be a negative gradient between human modified environments and prevalence of *P. falciparum* among anophelines (specifically *An. cruzii*) in this region. This new hypothesis shall be tested in a future and properly designed study.

Notwithstanding, all infected anophelines were collected in Shannon traps, which were not used in landscapes 1-B and 1-C (predominantly natural vegetation intermixed with rural areas). In contrast, CDC-LTs collected only 76 female anophelines, representing only 8.3% of the total captured. Half (38/76) of the CDC-LT-collected *Anopheles* were tested, according to the criteria defined before, and no specimen was positive for either *P. vivax* or *P. falciparum* DNA (power of the test = 0.87, considering the overall prevalence of 5.2%). Maybe this is because CDC-LT captures females not searching for a blood meal and those are predominantly young and nulliparous ones in the natural environment.

## Discussion

Results of the present study showed that *P. falciparum* and *P. vivax* are present and infect anophelines in forest-fragmented areas of the southeastern Atlantic forest where bromeliads are common and dense [36]. Lutz [1] in 1903 proposed the involvement of *Kerteszia* species (*An. cruzii* identified as *An. lutzi*) in the epidemiology of malaria in the southeastern Atlantic forest. Later, Downs and Pittendrigh [37] proposed the term “bromeliad-malaria” for the transmission involving species of the subgenus *Kerteszia*. In the following years, the bromeliad-malaria explanation was largely adopted by malariologists, and thus became a paradigm to explain the dynamics of residual malaria that still occur in areas of the Atlantic forest where *Anopheles* (*Kerteszia*) species are abundant and are primary vectors of *P. vivax* and *P. malariae* [2,3]. The larval habitats of the majority of the species in subgenus *Kerteszia* are bromeliad phytotelmata [25,38], except for *Anopheles bambusicolus* that is associated with the internodes of bamboo plants [38]. *Anopheles bellator*, *An. cruzii*, and *An. homunculus* are the most important vectors of *P. vivax* involved in the dynamics of the bromeliad-malaria in the Atlantic forest [39].

In the present study, however, results of *Anopheles* mosquitoes captured in seven landscapes, which have been distinctly modified by human activities, revealed a high prevalence of *An. cruzii* infected with *P. falciparum*, contrasting with a low proportion of infection by *P. vivax*. In addition, other species of the subgenus *Nyssorhynchus* of *Anopheles* were found infected with *P. falciparum* (*An. triannulatus*, *An. strodei* and *An. galvaoi*). The high prevalence in *An. cruzii* and those findings of *An. galvaoi*, *An. triannulatus* and *An. strodei* infected with *P. falciparum* represent, therefore, a challenge to the bromeliad-malaria paradigm. The found of *An. cruzii* as a potential vector of *P. falciparum*, and that species of another subgenus of *Anopheles* also may be involved in the malaria dynamics, in landscapes with different patterns of intermixed natural and human-modified environments of the southeastern Atlantic forest, suggest that residual malaria transmission in that region is complex and challenging. Therefore, malaria dynamics in that region may result from simultaneous cycles involving a range of anopheline species and, likely, monkeys as reservoirs.

Recent studies suggest that the bromeliad-malaria paradigm alone cannot explain the residual malaria in the Atlantic forest. For instances, there are several evidences of complex biological cycles involving different *Plasmodium* species and anopheline vectors. Duarte *et al*. [10] found that *An*. (*Nys*.) *lutzi* and *An. triannulatus* from anthropic zones were infected with *P. vivax*; *An. strodei* from transition zones (areas between sylvatic and anthropic zones) was positive for *P. malariae* and *An. cruzii* from sylvatic and anthropic regions tested positive for *P. vivax* and *P. malariae*. Similarly, Neves *et al*. [20] demonstrated that specimens of *An*. (*Ano*.) *fluminensis*, *An*. (*Ano*.) *pseudomaculipes*/*maculipes* and *An. cruzii* captured in a coastal area of an Atlantic forest reserve were infected with *P. vivax* and *P. malariae*. In addition, *P. falciparum* DNA has been reported in asymptomatic human residents in the mountainous regions of the Atlantic forest, in Espírito Santo state [18]. Furthermore, Yamasaki *et al*. [19] hypothesized that interacting human and zoonotic cycles of malaria transmission, including simians as potential reservoirs, occur in the Atlantic forest region.

Additionally, in the present work *Plasmodium*-infected anophelines were found in three out of seven landscapes composed of intermixed natural and human-modified environments. It should be mentioned here, however, that the sample sizes obtained in the negative landscapes were not large enough to exclude the possibility of *P. falciparum* circulation in the areas. These results also suggest a possible gradient between human environment modification and the occurrence of malaria. For instance, landscape 5, with a forest/rural environment ratio of 3:1, had the highest proportion of *P. falciparum*-infected anophelines (8.7% of *An. cruzii*, significantly higher than expected by chance); landscape 1-A (forest/rural ratio of 1.9:1) had 2.2% of *Nyssorhynchus* species infected with *P. falciparum*, an outcome expected by chance; finally, landscape 3 (with forest/rural ratio of 0.8:1) had a lower than expected proportion of *P. falciparum*-infected anophelines (0.9% of infected *Nyssorhynchus* species). In addition, landscape 5 is located in the same geographical region of São Paulo state where Maselli *et al.* [21] found a surprisingly high prevalence of subclinical *P. falciparum* infection among asymptomatic blood donors. Previous work by [40-42] also suggests that landscapes intermixing natural and human modified environments may favor malaria transmission.

The occurrence of major human infectious diseases has long been related to landscape modification, particularly by agriculture, which suggests that human modification of natural environments significantly increases the risk of infectious disease outbreaks, causing profound changes in the transmission dynamics of such infections, and possible evolutionary modifications in the pathogens [43]. Recent evidence on the possible origin of human *P. falciparum* in gorillas demonstrates the importance of such evolutionary pathogen modifications in response to evolving malaria transmission dynamics [44-47]. Krief *et al*. [48] proposes that *P. falciparum* may have evolved as a species in *Pan paniscus* (bonobos) and subsequently colonized humans by a host-switch. In addition, based on results of phylogenetic analysis, they assumed that *Pan troglodytes* (chimpanzees) and bonobo primates can act as reservoirs for all *Plasmodium* species to which humans are susceptible. On the other hand, the high prevalence of *P. falciparum* subclinical infections in blood donors living in forested areas of São Paulo [21] and the present findings of a high prevalence of *P. falciparum* in infected *An. cruzii* and other *Anopheles* (*Nyssorhynchus*) species in the same region, possibly indicate that this *Plasmodium* species may have adapted to monkeys as main reservoirs, in the Atlantic forest. Additional evidence supporting this hypothesis is the high genetic similarity between the DNA sequences from positive mosquitoes captured in Atlantic forest remains in the present study with those previously obtained from wild monkeys in Rondônia and deposited in the GenBank [35]. Of course, further evidence such as identification and genomic characterization of the parasites in wild monkeys of the Atlantic forest remains and the completion of socio-ecological surveys specifically designed to test such hypothesis are necessary to confirm these preliminary findings.

## Conclusions

The present study adds new and strong evidence to support the hypothesis of coexisting cycles of enzootic and human malaria, determining the residual malaria transmission dynamics in areas of the southeastern Atlantic forest. It also indicates that the bromeliad-malaria paradigm is not the only explanation for the dynamics of residual malaria transmission in the southeastern Brazilian Atlantic forest. Perhaps more important than this, is the recognition of the need for future studies focusing on both the evolutionary origin of *P. falciparum* that is circulating among different *Anopheles* species in the Atlantic forest and on the effects of different gradients of intermixed forest and human modified environments on malaria transmission in that region.

## Additional files

Additional file 1:
**Field design.** Schematic illustration representing types and position in the landscape of anopheline collection traps: 1) CDC-LT in the canopy in forest, 2) CDC-LT on the ground in forest, 3) CDC-LT in the canopy in forest margin, 4) CDC-LT on the ground in forest margin, 5) CDC-LT on the ground in open area, 6) CDC-LT with Octenol and CO_2_, and 7) Shannon trap. Trap drawings are out of scale and were modified from [25], Shannon, and [49], CDC-LT. Additional file 2:
**CDC-LT and Shannon traps utilized for field collections per landscape, Atlantic forest, Brazil, August-November 2012.**
Additional file 3:
**Successful amplification of** ***Plasmodium*** **species in** ***Anopheles*** **species per type of PCR method, Atlantic forest, Brazil, August-November 2012.**
Additional file 4:
**Description of the sequenced** ***Plasmodium*** **-infected** ***Anopheles*** **species, Atlantic forest, Brazil, August-November 2012.**

## Abbreviations

BLAST: Basic local alignment search tool
*COI*: Cytochrome oxidase 1
CSP: Circumsporozoite protein
DNA: Deoxyribonucleic acid
EDTA: Ethylenediamine tetraacetic acid
GIS: Geographic information system
Landsat TM: Land remote sensing satellite thematic mapper
PCR: Polymerase chain reaction
