# Supplementary figures and images for: Plasmodium falciparum in the southeastern Atlantic forest: a challenge to the bromeliad-malaria paradigm?
Source: Malar J. 2015 Apr 25;14:181. doi: 10.1186/s12936-015-0680-9 (PMC4417526; doi:10.1186/s12936-015-0680-9)

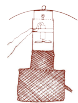

CDC-LT

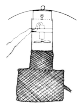

CDC-LT+Octenol+CO<sub>2</sub>

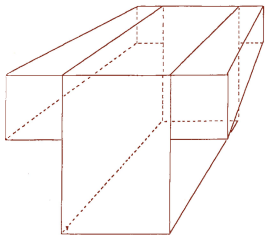

Shannon trap

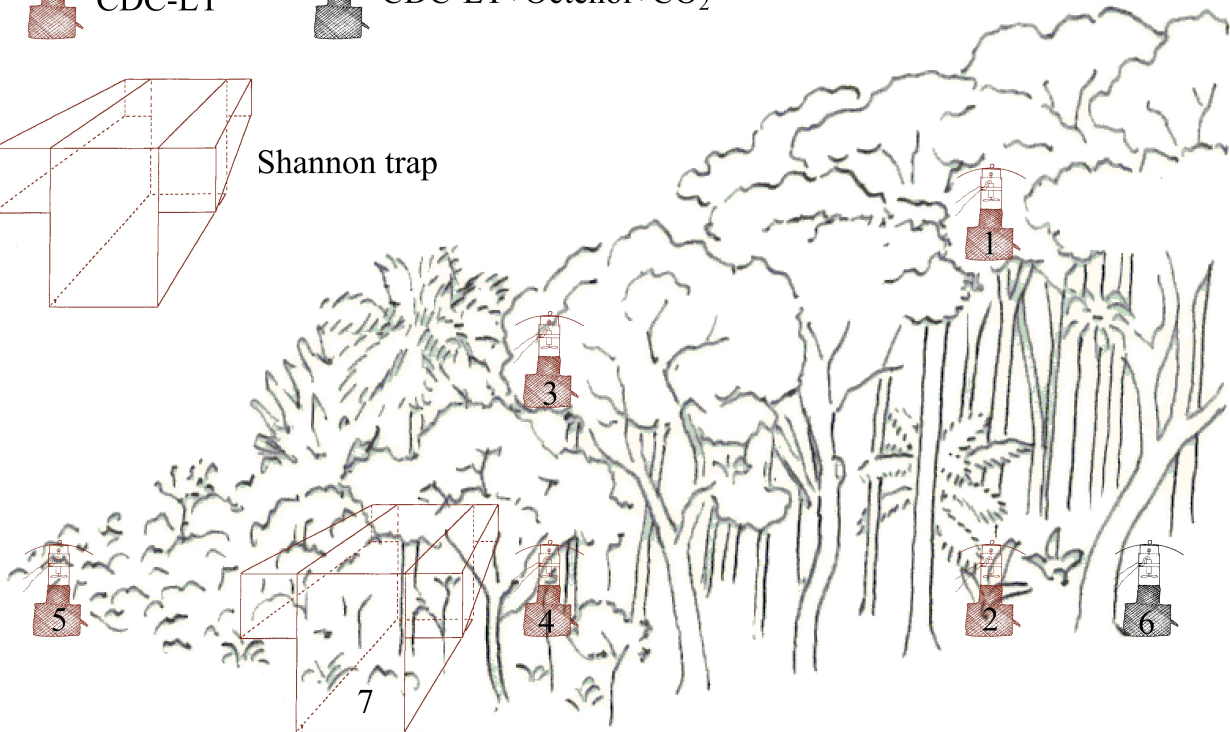

Supplement: Additional file 1: — Field design. Schematic illustration representing types and position in the landscape of anopheline collection traps: 1) CDC-LT in the canopy in forest, 2) CDC-LT on the ground in forest, 3) CDC-LT in the canopy in forest margin, 4) CDC-LT on the ground in forest margin, 5) CDC-LT on the ground in open area, 6) CDC-LT with Octenol and CO2, and 7) Shannon trap. Trap drawings are out of scale and were modified from [25], Shannon, and [49], CDC-LT. [file 12936_2015_680_MOESM1_ESM.pdf]
