# Supplementary material for: Plasmodium falciparum in the southeastern Atlantic forest: a challenge to the bromeliad-malaria paradigm?
Source: Malar J. 2015 Apr 25;14:181. doi: 10.1186/s12936-015-0680-9 (PMC4417526; doi:10.1186/s12936-015-0680-9)
Supplement: Additional file 2: — CDC-LT and Shannon traps utilized for field collections per landscape, Atlantic forest, Brazil, August-November 2012. [file 12936_2015_680_MOESM2_ESM.pdf]

Additional file 2 CDC-LT and Shannon traps utilized for field collections per landscape, Atlantic forest, Brazil, August-November 2012

Landscape	Date of collection	Trap (sampling effort)
1-A	22 Aug 2012	CDC-LT (72-hours)
1-A	23 Aug 2012	CDC-LT (72-hours)
1-A	17-18 Oct 2012	Shannon trap (12 hours)
1-B	13 Aug 2012	CDC-LT (72-hours)
1-B	14 Aug 2012	CDC-LT (72-hours)
1-C	15 Aug 2012	CDC-LT (72-hours)
1-C	16 Aug 2012	CDC-LT (72-hours)
2	20 Aug 2012	CDC-LT (72-hours)
2	21 Aug 2012	CDC-LT (72-hours)
2	12-13 Sept 2012	Shannon trap (12 hours)
3	29 Aug 2012	CDC-LT (72-hours)
3	30 Aug 2012	CDC-LT (72-hours)
3	24-25 Oct 2012	Shannon trap (12 hours)
4	27 Aug 2012	CDC-LT (72-hours)
4	28 Aug 2012	CDC-LT (72-hours)
4	19-20 Sept 2012	Shannon trap (12 hours)
5	28 Nov 2012	Shannon trap (3 hours)
