# Supplementary material for: Plasmodium falciparum in the southeastern Atlantic forest: a challenge to the bromeliad-malaria paradigm?
Source: Malar J. 2015 Apr 25;14:181. doi: 10.1186/s12936-015-0680-9 (PMC4417526; doi:10.1186/s12936-015-0680-9)
Supplement: Additional file 3: — Successful amplification of Plasmodium species in Anopheles species per type of PCR method, Atlantic forest, Brazil, August-November 2012. [file 12936_2015_680_MOESM3_ESM.pdf]

Additional file 3 Successful amplification of *Plasmodium* species in *Anopheles* species per type of PCR method, Atlantic forest, Brazil, August-November 2012

Species	*Plasmodium*	Successful amplification from real-time PCR	Successful amplification from conventional PCR	*Plasmodium* DNA sequencing
An. cruzii	*P. falciparum*	Yes	Yes	Yes
An. cruzii	*P. falciparum*	Yes	Yes	Yes
An. cruzii	*P. falciparum*	Yes	Yes	Yes
An. cruzii	*P. falciparum*	Yes	No	No
An. cruzii	*P. vivax*	Yes	No	No
An. cruzii	*P. vivax*	Yes	No	No
An. cruzii	*P. vivax*	Yes	No	No
An. cruzii	*P. vivax*	Yes	Yes	Yes
An. cruzii	*P. falciparum*	Yes	Yes	No
An. cruzii	*P. falciparum*	Yes	Yes	Yes
An. cruzii	*P. falciparum*	Yes	No	No
An. cruzii	*P. falciparum*	Yes	Yes	Yes
An. cruzii	*P. falciparum*	Yes	Yes	Yes
An. cruzii	*P. falciparum*	Yes	Yes	Yes
An. cruzii	*P. falciparum*	Yes	Yes	Yes
An. cruzii	*P. falciparum*	Yes	Yes	Yes
An. cruzii	*P. falciparum*	Yes	Yes	Yes
An. cruzii	*P. falciparum*	Yes	No	No
An. cruzii	*P. falciparum*	Yes	Yes	Yes
An. cruzii	*P. falciparum*	Yes	Yes	Yes
An. cruzii	*P. falciparum*	Yes	Yes	Yes
An. cruzii	*P. falciparum*	Yes	Yes	Yes
An. triannulatus	*P. falciparum*	Yes	No	No
An. strodei	*P. falciparum*	Yes	No	No
An. galvaoi	*P. falciparum*	Yes	Yes	Yes
