# Supplementary material for: Plasmodium falciparum in the southeastern Atlantic forest: a challenge to the bromeliad-malaria paradigm?
Source: Malar J. 2015 Apr 25;14:181. doi: 10.1186/s12936-015-0680-9 (PMC4417526; doi:10.1186/s12936-015-0680-9)
Supplement: Additional file 4: — Description of the sequenced Plasmodium -infected Anopheles species, Atlantic forest, Brazil, August-November 2012. [file 12936_2015_680_MOESM4_ESM.pdf]

Additional file 4 Description of the sequenced *Plasmodium*-infected *Anopheles* species, Atlantic forest, Brazil, August-November 2012

**Species**	**Date**	**Location**	**GenBank Access number**
An. cruzii	28 Nov 2012	Tapiraí, landscape 5	KM507293
An. cruzii	28 Nov 2012	Tapiraí, landscape 5	KM507294
An. cruzii	28 Nov 2012	Tapiraí, landscape 5	KM507295
An. cruzii	28 Nov 2012	Tapiraí, landscape 5	KM507296
An. cruzii	28 Nov 2012	Tapiraí, landscape 5	KM507297
An. cruzii	28 Nov 2012	Tapiraí, landscape 5	KM507298
An. cruzii	28 Nov 2012	Tapiraí, landscape 5	KM507299
An. cruzii	28 Nov 2012	Tapiraí, landscape 5	KM507300
An. cruzii	28 Nov 2012	Tapiraí, landscape 5	KM507301
An. cruzii	28 Nov 2012	Tapiraí, landscape 5	KM507302
An. cruzii	28 Nov 2012	Tapiraí, landscape 5	KM507303
An. cruzii	28 Nov 2012	Tapiraí, landscape 5	KM507304
An. cruzii	28 Nov 2012	Tapiraí, landscape 5	KM507305
An. cruzii	28 Nov 2012	Tapiraí, landscape 5	KM507306
An. cruzii	28 Nov 2012	Tapiraí, landscape 5	KM507307
An. cruzii	28 Nov 2012	Tapiraí, landscape 5	KM507308
An. cruzii	28 Nov 2012	Tapiraí, landscape 5	KM507309
An. cruzii	28 Nov 2012	Tapiraí, landscape 5	KM507310
An. cruzii	28 Nov 2012	Tapiraí, landscape 5	KM507311
An. cruzii	28 Nov 2012	Tapiraí, landscape 5	KM507312
An. cruzii	28 Nov 2012	Tapiraí, landscape 5	KM507313
An. cruzii	28 Nov 2012	Tapiraí, landscape 5	KM507314
An. triannulatus	18 Out 2012	Esteiro do Morro, landscape 1-A	KM507315
An. strodei	18 Out 2012	Esteiro do Morro, landscape 1-A	KM507316
An. galvaoi	25 Out 2012	Eldorado, landscape 3	KM507317
